# Fallacies and Possible Remedies of the SYNTAX Score

**DOI:** 10.1155/2020/8822308

**Published:** 2020-12-15

**Authors:** Yong-Ming He, Li Shen, Jun-Bo Ge

**Affiliations:** ^1^Division of Cardiology, The First Affiliated Hospital of Soochow University, Suzhou, Jiangsu, China; ^2^Department of Cardiology, Zhongshan Hospital, Fudan University, Shanghai Institute of Cardiovascular Diseases, Shanghai, China

## Abstract

Quite a few studies have revealed the clinical values regarding the outcome predictions in the cohort of the Synergy between Percutaneous Coronary Intervention with Taxus and Cardiac Surgery (SYNTAX) trial and decision-making with the SYNTAX score. The Evaluation of Xience Everolimus-Eluting Stent Versus Coronary Artery Bypass Surgery for Effectiveness of Left-Main Revascularization (EXCEL) and Nordic-Baltic-British left main revascularization (NOBLE) studies are the largest international randomized studies so far, comparing percutaneous coronary intervention (PCI) and coronary artery bypass graft (CABG) in the treatment of left main coronary artery disease. Unfortunately, both studies failed to validate the value of the SYNTAX score in the selection of revascularization strategies for patients with coronary artery diseases (CAD).. This scenario prompted us to reconsider the inherent fallacies of the SYNTAX score in its derivation. We pointed out eight fallacies for the SYNTAX score in this paper. A recently developed Coronary Artery Tree description and Lesion EvaluaTion (CatLet) score, available at http://www.catletscore.com, a novel angiographic scoring system, could be the remedies for the SYNTAX score.

## 1. Introduction

The Synergy between Percutaneous Coronary Intervention with Taxus and Cardiac Surgery (SYNTAX) score is an angiographic tool to grade the complexity and severity of coronary artery disease (CAD) [1]. Quite a few studies have revealed the clinical values regarding the outcome predictions in the cohort of the SYNTAX trial and the decision-making with the SYNTAX score [2–4]. The guideline gives a class I or IIa indication for percutaneous coronary intervention (PCI) in patients with significant left main disease and low or intermediate SYNTAX score [5]. The Evaluation of Xience Everolimus-Eluting Stent Versus Coronary Artery Bypass Surgery for Effectiveness of Left-Main Revascularization (EXCEL) and Nordic-Baltic-British left main revascularization (NOBLE) studies are the largest international randomized studies so far, comparing PCI and coronary artery bypass graft (CABG) in the treatment of left main coronary artery disease [6, 7]. Unfortunately, both studies failed to validate the value of the SYNTAX score in the selection of revascularization strategies for CAD patients. In the NOBEL trial, a substantially better outcome was still observed in the low SYNTAX score group undergoing CABG; In the EXCEL trial, 4-year mortality was comparable between PCI and CABG in both low (≤22) and intermediate [8–17] anatomical SYNTAX scores ((0.69 (95% prediction interval (PI): 0.34–1.45) vs. 0.93 (95% PI: 0.53–1.62)), without significant differences, in contrast with the SYNTAX trial [6, 7, 18, 19]. These studies challenged the original intention of the SYNTAX score at least in part, selecting optimal strategies for PCI or CABG [7, 20, 21]. In the PCI arm in the NOBEL trial, the SYNTAX score also failed to predict adverse outcomes, different from the SYNTAX trial [18, 19]. These conflicting results regarding the value of the outcome prediction with the SYNTAX score reminded us of its inherent fallacies in the derivation.

## 2. Oversimplified Dichotomization of the Coronary Circulation Pattern into Right or Left Dominance

**Figure 1:**
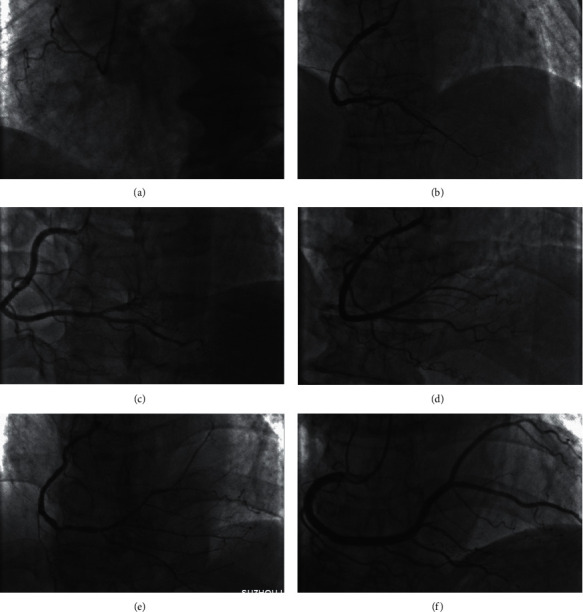
Failures in reflection of the variability in RCA blood supply to the LV in the SYNTAX score. (a) The RCA did not supply blood to the LV, which was defined as left dominant in the SYNTAX score. (b)–(f) The RCA, varying greatly in size, supplied blood to the LV, which was defined as right dominant and was assigned the same weighting in the SYNTAX score. LV = left ventricle; RCA = right coronary artery.

In the general population, the right coronary artery (RCA) supplying blood to the territory of the left ventricle (LV) should be a continuous spectrum with two extremes at either side: the RCA does not supply blood to the LV with a resulting dominant left circumflex (LCX) or the RCA supplies blood to most portion of the LV with a resulting small LCX. Thus, any attempt to simply dichotomize the RCA size is not wise and will obviously deviate from the clinical practice. Figures 1(a)–1(f) present progressive changes in RCA sizes. According to the SYNTAX score, Figures 1(b)–1(f) are all defined as right dominant, and their weightings are invariably 1.0. As a matter of fact, it is difficult to understand that the RCA in Figure 1(b) contributes equally as in Figure 1(f) in terms of blood supply to the LV. It is also not surprising that it is difficult to find vessels to be appropriately named as 16a, 16b, or 16c in the SYNTAX score calculation.

## 3. Mixed-Up Values from the Stenosis Lesion and Its Adverse Characteristics

The SYNTAX score encompasses two components: one is from the stenosis lesion and the other is from its adverse characteristics, such as bifurcation, trifurcation, angulation, thrombus, and calcification. For the stenosis lesion, the score is the product of a weighting factor of a diseased vessel and its stenosis degree; for those adverse characteristics, the score values are directly assigned. It is obviously inappropriate to mix up these two scores of different origins with different implications, which are required to be investigated separately.

## 4. Violation of the Law of Flow Conservation in the Weighting Assignment

According to the law of flow conservation [22], a parent vessel equals the sum of its daughter vessels in terms of blood flow. In the right dominance, the RCA gives rise to the posterior descending artery (PDA) and posterolateral vessels (PLVs); therefore, the proximal, mid, or distal RCA should equal the sum of the PDA and PLVs in terms of blood flow. Correspondingly, the weighting factor of the proximal, mid, or distal RCA should equal the sum of the PDA and PLVs. Unfortunately, in the SYNTAX score, the weighting of the proximal, mid, or distal RCA is 1.0, the weighting of the PDA, a branch from the RCA, is surprisingly 1.0, and the weighting of PLVs, also branches from the RCA, is surprisingly 1.5 (0.5 + 0.5 + 0.5), violating the law of flow conservation and leading to logic errors as shown in Table 1 [1].

## 5. The Nomenclature-Based Weighting Assignment of the Coronary Tree Segments

In the SYNTAX score, the weighting assignment to the coronary tree segments remains to be essentially nomenclature-based although their importance has been partly considered. The intermediate branch has been invariably assigned 1.0 in right-dominant circulation in the SYNTAX score. In most circumstances, however, patients had intermediate branches of different sizes, and this invariable assignment of 1.0 failed in reflecting the variability in the intermediate branches.

## 6. Arbitrary Value Assignment for Those Adverse Characteristics

Unfortunately, we, as of today, have failed to find the evidence based on which the score values are assigned for those adverse characteristics [1]. Value assignments for adverse characteristics are arbitrary in the SYNTAX score. Actually, most adverse characteristics except the calcification have not predicted clinical outcomes for CAD patients [23–28]. Therefore, independent studies are required to identify different contributions to outcome predictions for individual adverse characteristics before we assign values to those adverse characteristics.

## 7. Lower Reproducibility of the SYNTAX Score

For ordered variables, the weighted kappa values were used to express the degree of agreement: slight, kappa values ranging 0.0∼0.2; fair, ranging 0.2∼0.4; moderate, ranging 0.4∼0.6; substantial, ranging 0.6∼0.8; almost perfect, ranging 0.8∼1.0 [29]. The SYNTAX trial investigators first reported a weighted kappa value of 0.45 for interobserver reproducibility on the global SYNTAX score and a weighted kappa value of 0.59 for intraobserver reproducibility [30]. Another independent study from the SYNTAX trial investigators also reported a moderate degree of agreement with a kappa value of 0.51 for intraobserver reproducibility according to the tertile analysis (<23, 23∼33, ≥33) on the global SYNTAX score [31]. Two studies from the non-SYNTAX trial team independently assessed the reproducibility of the SYNTAX score and found that the weighted kappa values were less than 0.60 for interobserver variability according to the tertile analysis (<23, 23∼33, ≥33), while the weighted kappa values for intraobserver variability were around 0.70. The interobserver agreement of on-site interventional cardiologists was initially at best fair (kappa = 0.33 [0.18, 0.44]) after basic training, improving to substantial or greater after advanced training (kappa = 0.76 [0.64, 1.00]). Despite advanced training, the on-site interventional cardiologists still underscored the number of lesions, bifurcations, and small-vessel disease, which resulted in a lower reproducibility than the core lab technicians [32].

## 8. SYNTAX  Score II Is Not a True Remedy for the Anatomical SYNTAX Score

SYNTAX score II performed better than the anatomical SYNTAX score in 4-year mortality predictions, demonstrating that the seven more predictors (age, creatinine clearance, left ventricular ejection fraction (LVEF), presence of unprotected left main coronary artery (ULMCA) disease, peripheral vascular disease, female sex, and chronic obstructive pulmonary disease (COPD)) had predicting values and that the anatomical SYNTAX score left much room to be improved [8]. Only 35% of the intermediate (50–70%) angiographic stenosis were hemodynamically relevant as defined by the fractional flow reserve (FFR) ≤0.80 [9]. In the ERACI (Estudio Randomizado Argentino Angioplastia versus Cirugia) risk score, modifying the basal and residual SYNTAX score calculation, the intermediate lesions (50∼69%), small vessels (1.5∼2.0 mm in diameter), and some adverse characteristics, such as bifurcation, trifurcation, and chronic total occlusion, were excluded from being scored. This simple exclusion yielded similar results as compared with the fractional flow reserve-guided revascularization [10–12]. The FFR-guided evaluation of lesions will help in the SYNTAX score calculation. It is unimaginable that the anatomical SYNTAX score needing seven more predictors to fix up is a good tool in clinical practice and that the seven predictors sufficed to construct a new model to replace the anatomical SYNTAX score. Actually, the parsimonious model, ACEF (age, creatinine, and LVEF), predicted clinical outcomes well, with reported concordance indices of 0.63∼0.81, similar to or better than the anatomical SYNTAX score [13–15]. Therefore, any remedies not remedying the anatomical SYNTAX score per se should not be considered as true remedies.

## 9. The Need for Adapting Modification

**Figure 2:**
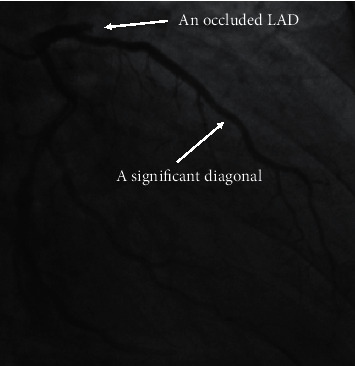
The need for adapting modification in the scoring of the LAD in the presence of a significant diagonal preceding the lesion. In the right anterior oblique 30°/caudal 20° view, the proximal LAD was totally occluded. A significant diagonal without a significant stenosis preceded the occlusive lesion. LAD = left anterior descending artery.

Within one segment, direct scoring will overestimate the severity of coronary artery disease in the presence of side branches preceding the main branch lesion regardless of their normal or diseased status. In Figure 2, the proximal LAD had an occlusive lesion and a significant diagonal. According to the SYNTAX score, this occlusive lesion will be directly scored as 3.5 × 5 = 17.5, which obviously overestimates the severity of the LAD because of the presence of the significant diagonal. Therefore, this occlusive lesion on the proximal LAD should be reasonably modified for 3.5 × 5 − 1.0 × 5 = 12.5.

## 10. Remedies for the SYNTAX Score

The Coronary Artery Tree description and Lesion EvaluaTion (CatLet) score, accommodating the variability in the coronary anatomy, has been a recently developed comprehensive angiographic scoring system aimed at assisting in the risk stratification of CAD patients and in the standardization of the angiographic data collection [16]. In the CatLet score, the lesion and the adverse angiographic characteristics are defined as in the SYNTAX score. The CatLet score had four characteristics: (1) the coronary circulation pattern is overall evaluated first before we proceed; (2) only a lesion ≥50% diameter stenosis in vessels >1.5 mm in diameter is scored and modified if appropriate; (3) the eight adverse angiographic characteristics pertinent to the lesion are not scored any more, but only qualitatively recorded instead; and (4) the diffuse disease/small vessels are given up to be evaluated, quantitatively or qualitatively. Most reproducibility studies did not report the kappa values regarding the diffuse disease/small vessels in the SYNTAX score [17, 30, 31, 33]; two studies reported that the kappa values regarding the diffuse disease/small vessels were less than 0.4, at best a fair degree of agreement even after advanced training [32], highlighting the difficulty in evaluating the diffuse disease/small vessels defined in the SYNTAX score. That is the reason why we give up to evaluate this angiographic characteristic. Regarding the eight adverse angiographic characteristics, their individual contributions to the outcome prediction need to be investigated separately from the lesion score. The calculator of the CatLet score and its elaborate tutorial are available at http://www.catletscore.com. The IE browser or Microsoft Edge is required to run this calculator.  Table 2 lists the differences and similarities with respect to criteria and definitions between the SYNTAX score and the CatLet score.

This novel CatLet angiographic scoring system has been described in full, following the following classifications and rules: the 17-myocardial segment model, the law of flow conservation, and the law of competitive blood supply [22, 34]. In the 17-myocardial segment model, the LV is approximately evenly divided into 17 segments. Each segment can be taken as 1 mass unit or 1 actual flow unit given that the actual myocardial blood flow is proportional to the muscle mass. The importance of a blood vessel depends on how many segments the blood vessel supplies rather than on the nomenclature of the blood vessel. Therefore, if the LAD supplies 7 segments, its weighting factor will be assigned 7.0. The weighting assignment to LCX and RCA follows the same rule. According to our new reclassification scheme, the RCA is progressively divided into 6 types: PDA zero, PDA only, small RCA, average RCA, large RCA, and super RCA; the LAD is progressively divided into 3 types: short, average, and long; and the Dx is progressively divided into 3 types: small, intermediate, and large, which together result in 54 types of the coronary circulation pattern [16]. A total of 54 types of the coronary circulation pattern are utilized to cover the variability of the coronary anatomy among individuals. The coursing and supplying territory are relatively easy to be identified for LAD, PDA, and Dx, but not for LCX on the angiograms. LCX is indirectly reflected by RCA, LAD, and Dx according to the law of competitive blood supply. Therefore, the first step to overall evaluate the coronary circulation pattern is achieved by the evaluation of LAD, PDA, and Dx, but not including LCX.

Our first validation study revealed that the CatLet score better predicted the clinical outcomes for acute myocardial infarction patients than the SYNTAX score [35]. Compared with the SYNTAX score, the CatLet score had a better discrimination and calibration for outcome prediction, which resulted in a better refined and balanced risk stratification with the CatLet score according to its tertile analysis (≤9, 10–14, and >15). Specifically, 6.2% of patients with MACCE and 6.6% of patients without MACCE were correctly reclassified from the SYNTAX tertiles into the CatLet score tertiles, respectively. Consequently, the category-free net reclassification improvement (NRI) was 37.2% (*z* = 2.65, *P*=0.008). NRIs for 4.3-year cardiac death and all-cause death were 35.5% (*P*=0.0249) and 31.8% (*P*=0.0316), respectively [35]. We also reported a satisfactory kappa value greater than 0.8, almost an excellent degree of agreement beyond the level of chance with the CatLet score [36].

Given that the CatLet score is derived based on the acknowledged classifications and rules, and its utility has been preliminarily validated in patients with acute myocardial infarction undergoing primary PCI. Therefore, the CatLet angiographic scoring system proposed recently may be the remedies for the SYNTAX score although we have to admit that the value of this novel angiographic scoring system warrants further validation in different CAD populations, such as left main disease and chronic total occlusion.

## Figures and Tables

**Table 1:** Weighting factor assignment to the RCA system for the right dominance in the SYNTAX score.

	Segment number	Weighting
1	RCA proximal	1.0
2	RCA mid	1.0
3	RCA distal	1.0
4	Posterior descending artery	1.0
16	Posterolateral branch from the RCA	0.5
16a	Posterolateral branch from the RCA	0.5
16b	Posterolateral branch from the RCA	0.5
16c	Posterolateral branch from the RCA	0.5

RCA = right coronary artery.

**Table 2:** Differences and similarities with respect to criteria and definitions between the SYNTAX score and CatLet score.

Items	Values	
Calculators	CatLet score	SYNTAX score
1. Underlying mechanism	Weighted matrix#	Leaman score
2. Dominance selection		
RCA dominance	6 types	2 types
Dx size	3 types	NA
LAD length	3 types	NA
3. Lesion definition	As in the SYNTAX	≥50% diameter stenosis on vessels >1.5 mm
4. Multiplication factor	As in the SYNTAX	5.0 for occlusion 2.0 for nonocclusion
5. Lesion types	6 types	2 types
Native nonocclusion (50–99% diameter reduction)	Yes	Yes
Native acute total occlusion (ATO)	Yes	Yes
Native chronic total occlusion (CTO)	Yes	Yes
In-stent nonocclusion	Yes	NA
In-stent ATO	Yes	NA
In-stent CTO	Yes	NA
6. Scoring modification	Yes if appropriate	No
7. Culprit vessels	Indicated	No
8. Trifurcation	Yes/no	Scored
9. Bifurcation	Yes/no	Scored
10. Aorta ostial lesion	Yes/no	Scored
11. Severe tortuosity	Yes/no	Scored
12. Length >20 mm	Yes/no	Scored
13. Heavy calcification	Yes/no	Scored
16. Thrombus	Yes/no	Scored
17. Diffuse disease/small vessels	Discarded	Scored

Notes: CatLet = Coronary Artery Tree description and Lesion EvaluaTion; SYNTAX = Synergy between Percutaneous Coronary Intervention with Taxus and Cardiac Surgery; RCA = right coronary artery; LAD = left anterior descending artery; Dx = diagonals; NA = not available. ^#^See reference [16].

## Data Availability

The data used to support the findings of this study are available at http://www.chictr.org.cn (unique identifiers: ChiCTR-POC-17013536).

## References

[1] Sianos G., Morel M. A., Kappetein A. P. (2005). The SYNTAX score: an angiographic tool grading the complexity of coronary artery disease. *EuroIntervention :Journal of EuroPCR in Collaboration with the Working Group on Interventional Cardiology of the European Society of Cardiology*.

[2] Wykrzykowska J. J., Garg S., Girasis C. (2010). Value of the SYNTAX score for risk assessment in the all-comers population of the randomized multicenter LEADERS (Limus eluted from a durable versus ERodable stent coating) trial. *Journal of the American College of Cardiology*.

[3] Garg S., Serruys P. W., Silber S. (2011). The prognostic utility of the SYNTAX score on 1-year outcomes after revascularization with zotarolimus- and everolimus-eluting stents. *JACC: Cardiovascular Interventions*.

[4] Cavalcante R., Sotomi Y., Mancone M. (2017). Impact of the SYNTAX scores I and II in patients with diabetes and multivessel coronary disease: a pooled analysis of patient level data from the SYNTAX, PRECOMBAT, and BEST trials. *European Heart Journal*.

[5] Neumann F. J., Sousa-Uva M., Ahlsson A. (2018). ESC/EACTS Guidelines on myocardial revascularization. *European Heart Journal*.

[6] Campos C. M., van Klaveren D., Farooq V. (2015). Long-term forecasting and comparison of mortality in the evaluation of the xience everolimus eluting stent vs. Coronary artery bypass surgery for effectiveness of left main revascularization (EXCEL) trial: prospective validation of the SYNTAX score II. *European Heart Journal*.

[7] Mäkikallio T., Holm N. R., Lindsay M. (2016). Percutaneous coronary angioplasty versus coronary artery bypass grafting in treatment of unprotected left main stenosis (NOBLE): a prospective, randomised, open-label, non-inferiority trial. *The Lancet*.

[8] Farooq V., van Klaveren D., Steyerberg E. W. (2013). Anatomical and clinical characteristics to guide decision making between coronary artery bypass surgery and percutaneous coronary intervention for individual patients: development and validation of SYNTAX score II. *The Lancet*.

[9] Van Belle E., Rioufol G., Pouillot C. (2014). Outcome impact of coronary revascularization strategy reclassification with fractional flow reserve at time of diagnostic angiography. *Circulation*.

[10] Haiek C., Fernández-Pereira C., Santaera O. (2017). Second vs. First generation drug eluting stents in multiple vessel disease and left main stenosis: two-year follow-up of the observational, prospective, controlled, and multicenter ERACI IV registry. First generation drug eluting stents in multiple vessel disease and left main stenosis: two-year follow-up of the observational, prospective, controlled, and multicenter ERACI IV registry. *Catheterization and Cardiovascular Interventions*.

[11] Rodriguez A. E., Fernandez-Pereira C., Mieres J. (2018). Lowering risk score profile during PCI in multiple vessel disease is associated with low adverse events: the ERACI risk score. *Cardiovascular Revascularization Medicine*.

[12] Rodriguez A. E., Fernandez-Pereira C., Mieres J., Mendoza J., Sartori F. (2017). Can we improve the outcomes of multivessel disease using modified SYNTAX and residual SYNTAX scores?. *Current Cardiology Reports*.

[13] Biondi-Zoccai G., Romagnoli E., Castagno D. (2012). Simplifying clinical risk prediction for percutaneous coronary intervention of bifurcation lesions: the case for the ACEF (age, creatinine, ejection fraction) score. *EuroIntervention*.

[14] Wykrzykowska J. J., Garg S., Onuma Y. (2011). Value of age, creatinine, and ejection fraction (ACEF score) in assessing risk in patients undergoing percutaneous coronary interventions in the “all-comers” LEADERS trial. *Circulation: Cardiovascular Interventions*.

[15] Ranucci M., Castelvecchio S., Menicanti L., Frigiola A., Pelissero G. (2009). Risk of assessing mortality risk in elective cardiac operations. *Circulation*.

[16] Xu M.-X., Teng R.-L., Teng R.-L. (2019). The CatLet score: a new coronary angiographic scoring tool accommodating the variable coronary anatomy for the first time. *Journal of Thoracic Disease*.

[17] Shiomi H., Tamura T., Niki S. (2011). Inter- and intra-observer variability for assessment of the synergy between percutaneous coronary intervention with TAXUS and cardiac surgery (SYNTAX) score and association of the SYNTAX score with clinical outcome in patients undergoing unprotected left main stenting in the real world. *Circulation Journal*.

[18] Mohr F. W., Morice M.-C., Kappetein A. P. (2013). Coronary artery bypass graft surgery versus percutaneous coronary intervention in patients with three-vessel disease and left main coronary disease: 5-year follow-up of the randomised, clinical SYNTAX trial. *The Lancet*.

[19] Serruys P. W., Morice M.-C., Kappetein A. P. (2009). Percutaneous coronary intervention versus coronary-artery bypass grafting for severe coronary artery disease. *New England Journal of Medicine*.

[20] Morice M.-C., Serruys P. W., Kappetein A. P. (2014). Five-year outcomes in patients with left main disease treated with either percutaneous coronary intervention or coronary artery bypass grafting in the synergy between percutaneous coronary intervention with taxus and cardiac surgery trial. *Circulation*.

[21] Park S.-J., Ahn J.-M., Kim Y.-H. (2015). Trial of everolimus-eluting stents or bypass surgery for coronary disease. *New England Journal of Medicine*.

[22] Ormiston J., Kassab G., Finet G. (2018). Bench testing and coronary artery bifurcations: a consensus document from the European bifurcation club. *EuroIntervention*.

[23] Campos C. M., Costa F., Garcia-Garcia H. M. (2015). Anatomic characteristics and clinical implications of angiographic coronary thrombus: insights from a patient-level pooled analysis of SYNTAX, RESOLUTE, and LEADERS Trials. *Circulation Cardiovascular Interventions*.

[24] Palmerini T., Genereux P., Caixeta A. (2011). Prognostic value of the SYNTAX score in patients with acute coronary syndromes undergoing percutaneous coronary intervention. *Journal of the American College of Cardiology*.

[25] Bourantas C. V., Zhang Y.-J., Garg S. (2015). Prognostic implications of severe coronary calcification in patients undergoing coronary artery bypass surgery: an analysis of the SYNTAX Study. *Catheterization and Cardiovascular Interventions*.

[26] Bourantas C. V., Zhang Y.-J., Garg S. (2014). Prognostic implications of coronary calcification in patients with obstructive coronary artery disease treated by percutaneous coronary intervention: a patient-level pooled analysis of 7 contemporary stent trials. *Heart*.

[27] Girasis C., Farooq V., Diletti R. (2013). Impact of 3-dimensional bifurcation angle on 5-year outcome of patients after percutaneous coronary intervention for left main coronary artery disease. *JACC: Cardiovascular Interventions*.

[28] Capodanno D., Di Salvo M. E., Cincotta G., Miano M., Tamburino C., Tamburino C. (2009). Usefulness of the SYNTAX score for predicting clinical outcome after percutaneous coronary intervention of unprotected left main coronary artery disease. *Circulation: Cardiovascular Interventions*.

[29] Bland J. M., Altman D. G. (1999). Measuring agreement in method comparison studies. *Statistical Methods in Medical Research*.

[30] Serruys P., Onuma Y., Garg S. (2009). Assessment of the SYNTAX score in the Syntax study. *EuroIntervention*.

[31] Garg S., Girasis C., Sarno G. (2010). The SYNTAX score revisited: a reassessment of the SYNTAX score reproducibility. *Catheterization and Cardiovascular Interventions : Official Journal of the Society for Cardiac Angiography & Interventions*.

[32] Généreux P., Palmerini T., Caixeta A. (2011). SYNTAX score reproducibility and variability between interventional cardiologists, core laboratory technicians, and quantitative coronary measurements. *Circulation: Cardiovascular Interventions*.

[33] Garot P., Tafflet M., Kumar S. (2012). Reproducibility and factors influencing the assessment of the SYNTAX score in the left main Xience study. *Catheterization and Cardiovascular Interventions*.

[34] Cerqueira M. D., Weissman N. J., Dilsizian V. (2002). Standardized myocardial segmentation and nomenclature for tomographic imaging of the heart. a statement for healthcare professionals from the cardiac imaging committee of the council on clinical cardiology of the American heart association. *Circulation*.

[35] Xu M. X., Ruddy T. D., Schoenhagen P. (2020). The CatLet score and outcome prediction in acute myocardial infarction for patients undergoing primary percutaneous intervention: a proof-of-concept study. *Catheterization and Cardiovascular Interventions*.

[36] Liu J. M., He Y., Teng R. L. (2020). Inter-and intra-observer variability for the assessment of coronary artery tree description and lesion EvaluaTion (CatLet^©^) angiographic scoring system in patients with acute myocardial infarction. *Chinese Medical Journal*.

